# Mental performance skills and athletic performance in combat athletes: the mediating role of mental toughness

**DOI:** 10.3389/fpsyg.2026.1848217

**Published:** 2026-06-19

**Authors:** Mehdi Duyan, İlker Günel, Fatma Özoğlu, Servet Reyhan, Talip Çelik, Gamze Ok, Mihriay Musa, Matteo Giuriato

**Affiliations:** 1 Faculty of Sports Sciences, İnönü University, Malatya, Türkiye; 2 Department of Physical Education and Sports, Osmaniye Korkut Ata University, Osmaniye, Türkiye; 3 Malatya Vocational School of Property Protection and Security, İnönü University, Malatya, Türkiye; 4 Institute of Health Sciences, Department of Physical Education and Sports, İnönü University, Malatya, Türkiye; 5 Department of Coaching Education, Faculty of Sports Sciences, Topkapı University, Istanbul, Türkiye; 6 Department of Education and Sport Sciences, Pegaso Telematic University, Naples, Italy

**Keywords:** athletic performance, combat sports, mental performance skills, mental toughness, structural equation modeling

## Abstract

**Introduction:**

Mental performance skills are recognized as critical for success in combat sports, yet the underlying mechanisms through which they influence performance remain poorly understood. This study examines the mediating role of mental toughness between mental performance skills and athletic performance within the framework of Conservation of Resources Theory.

**Method:**

This quantitative research employed a correlational survey model. The study included 470 combat sport athletes (243 male, 227 female) from various universities in Türkiye. Participants completed the Mental Performance Skills Scale, the Mental Toughness Inventory, and the Athletic Performance Scale for Combat Athletes. Data were analyzed using Pearson correlation analysis and Structural Equation Modeling. Model fit indices were calculated as CFI = 0.983, TLI = 0.981, RMSEA = 0.08, and SRMR = 0.07.

**Results:**

Correlation analysis revealed positive and significant correlations among all variables (*r* = 0.394–0.445, *p* < 0.001). According to Cohen’s conventions, these values represent medium-to-large effect sizes. Structural equation modeling results indicated that mental performance skills had significant direct effects on athletic performance (*β* = 0.353, *p* < 0.001) and on mental toughness (*β* = 0.483, *p* < 0.001), and that mental toughness significantly predicted athletic performance (*β* = 0.361, *p* < 0.001). Bootstrap analysis confirmed that mental toughness served as a significant mediator, as the indirect effect [*β* = 0.174, 95% CI (0.111, 0.204)] did not contain zero. The direct effect (*β* = 0.353, *p* < 0.001) remained significant after accounting for the indirect pathway.

**Conclusion:**

In conclusion, mental performance skills strengthen mental toughness, and this enhanced mental toughness plays a significant mediating role in improving athletic performance. Structured mental skills training can improve not only competition results but also fundamental psychological capacities such as stress management and coping with challenging conditions.

## 1 Introduction

Sport performance in disciplines such as combat sports, which involve high competition, physical contact, and instantaneous decision-making pressure, is closely related not only to physical capacity but also to athletes’ psychological skills and stress management abilities (Piskorska et al., 2016; Slimani et al., 2018; Mojtahedi et al., 2023). Combat sports are among the sports disciplines characterized by high individual responsibility, low error tolerance, and intense cognitive and emotional pressure during competition (Slimani and Che, 2016).

Unlike many sports, combat sports are characterized by physical confrontation, high injury risk, and immediate performance feedback in the form of winning or losing, placing intense stress on athletes’ psychological resources (Soundara Pandian et al., 2023; Kumar and Kumar, 2024; Quan et al., 2025). Athletes exposed to intense stress, anxiety, and depressive symptoms before or during competition exhibit marked distractibility and difficulty focusing, which disrupts their ability to make quick and accurate decisions, thereby limiting their performance potential (Aksoy, 2019; Chen et al., 2020; Ning et al., 2022). Accordingly, one of the applications of sport psychology is to help improve performance, learn skills, and apply them (Khodabandelou and Salehian, 2023).

Mental performance skills (MPS), including goal setting, imagery, self-talk, arousal regulation, and focus, are considered trainable psychological tools that athletes use to optimize their competitive state and perform under pressure (Birrer and Morgan, 2010; Bühlmayer et al., 2017; Vealey, 2019). MPS are defined as learnable and developable psychological strategies that support performance, such as goal setting, imagery, attention control, self-talk, and relaxation (Vealey, 2024). Recent research has shown that these skills contribute to athletes’ more consistent and effective performance in competitive environments (Bühlmayer et al., 2017; Röthlin and Birrer, 2020). Therefore, mental training skills are considered an important tool for protecting athletes’ psychological health and enhancing their performance. Mental training skills have been reported to improve emotion regulation abilities (Hut et al., 2021), increase self-efficacy, alleviate anxiety and depressive symptoms (Röthlin and Birrer, 2020; Yuan et al., 2021; Lu and Xu, 2023; Aulia et al., 2024), and support performance (Yue et al., 2025). Additionally, mental training skills have been reported to reduce injury anxiety and enhance optimal performance in athletes (Duyan et al., 2025). Regarding their theoretical foundations, symbolic learning theory suggests that individuals can create a “schema” in their minds to perform a specific behavior. Psychoneuromuscular theory posits that mental imagery activates the motor cortex, producing neuromuscular activation similar to actual physical performance (Fortes et al., 2016). Mental training has been observed to improve physiological processes by providing effective control over the autonomic nervous system (Altıntaş and Akalan, 2008). In addition to their physical and psychological benefits, combat sports also involve certain risks that can negatively affect athletes’ performance. These include excessive training load (Slimani and Che, 2016; Andreato et al., 2022; Lakicevic et al., 2022), injury anxiety (Mojtahedi et al., 2023; Predoiu et al., 2024; Quan et al., 2025), weight pressure (Artioli et al., 2016; Rossi et al., 2022; Lakicevic et al., 2024), and intense competitive stress (Mojtahedi et al., 2023; Lakicevic and Panfilova, 2025; Quan et al., 2025).

Combat sports such as taekwondo, karate, and kickboxing require high levels of physical capacity (speed, agility, strength) and psychological preparation. Psychological factors such as self-efficacy, motivation, and MT have long been recognized as essential elements for performance excellence (Slimani and Che, 2016). In this context, the role of psychological processes in understanding the performance of combat athletes is gaining increasing importance. Consequently, with the growing emphasis on psychological processes, the field of exercise and sport psychology has focused on identifying and examining the mental skills underlying success in these high-risk environments (Andrade et al., 2021; Zhao et al., 2024; Quan et al., 2025).

However, it has been suggested that the effects of MPS on AP are not always direct and that this relationship may be shaped by certain psychological mechanisms. One prominent mechanism in this regard is MT, which refers to the athlete’s capacity to maintain performance and recover despite pressure, stress, and adversity (Gucciardi et al., 2009, 2015; Cowden, 2016). From a theoretical perspective, it is possible that MPS enhance sport performance indirectly by strengthening athletes’ mental toughness, rather than directly. The sudden stressors, physical contact, and competitive pressure encountered in combat sports place MT at the center of performance (Slimani and Che, 2016; Slimani et al., 2016). MT plays a decisive role in maintaining performance under intense pressure, recovering from injury, and achieving ultimate competitive success (Lakicevic and Panfilova, 2025). MT is defined as an important psychological resource for overcoming difficult and challenging conditions (Hsieh et al., 2024). Combat athletes, constantly confronted with the physical and psychological stressors inherent in training and competition, consider mental toughness an indispensable psychological resource (Sarkar and Fletcher, 2014; Predoiu et al., 2024). MT plays an important role in sustaining the performance of combat athletes and protecting their psychological well-being (Mojtahedi et al., 2023; Lakicevic and Panfilova, 2025).

Within the framework of Conservation of Resources Theory (Hobfoll, 1989), individuals strive to protect their valuable psychological resources (attention, energy, self-confidence) and acquire new resources by investing in them. In this context, MPS (goal setting, imagery, focus) function as “resource conservation strategies”, while mental toughness can be conceptualized as a more stable and resilient “accumulated resource” resulting from the regular use of these strategies. This accumulated resource ultimately leads to a tangible “resource gain” in the form of performance. Although COR theory has been extensively applied in occupational and health psychology, it has seen scant use in sport psychology (Nahum, 2024). Its application in combat sports remains particularly limited (Lakicevic and Panfilova, 2025). According to COR theory, resource gain spirals occur when initial resource investment leads to resource accumulation, which facilitates further resource acquisition (Hobfoll, 2002). Recent empirical studies have confirmed that psychological resources enhance MT through COR-based mechanisms (Xu and Tian, 2025).

Despite growing interest in psychological processes in combat sports, three critical gaps remain in the literature. First, no study has simultaneously tested the integrated MPS → MT → AP mediation model in combat sports. While Demir et al. (2025) used structural equation modeling to examine the relationships among mental toughness, sport imagery, anxiety, and athletic performance, their study had several differences from the present investigation. Their sample consisted of both team and individual sport athletes, whereas the present study focuses exclusively on combat sport athletes. Additionally, their study did not include mental performance skills (MPS) as a predictor variable; rather, mental toughness served as the independent variable. Although sport imagery is a component of MPS, it does not fully represent the broader construct of MPS, which includes goal setting, self-talk, arousal regulation, focus, and other trainable psychological skills. In contrast, the present study conceptualizes MPS as a holistic construct and tests its effects on athletic performance specifically in combat sport athletes. Furthermore, their athletic performance measure was not specific to combat sports, whereas the present study employs a combat-sport-specific athletic performance scale (Bingöl and Yildiz, 2021).

Second, although mental toughness is recognized as a critical resource in combat sports (Mojtahedi et al., 2023; Lakicevic and Panfilova, 2025), its mediating role between MPS and AP has not been directly tested. Mojtahedi et al. (2023) demonstrated that mentally tough combat athletes experience lower levels of cognitive and somatic anxiety, but they did not examine how mental toughness develops from psychological skills training or whether it mediates the MPS-AP relationship. Similarly, Lakicevic and Panfilova (2025) critically examined the literature on mental toughness in combat sports and concluded that while psychological skills training can enhance self-reported mental toughness, the specific mechanisms through which psychological skills translate into performance outcomes remain untested in combat sports populations. This gap in the literature underscores the need for research that explicitly examines how and why mental skills translate into performance gains via mental toughness.

Third, existing studies that have tested mediation models in sport contexts have focused on different outcome variables or different populations. For instance, Zhang and Yu (2025) found that MT mediates the relationship between sports psychological skills and athlete burnout, but their dependent variable was burnout rather than AP. Moreover, their sample was not limited to combat sports athletes. Hsieh et al. (2024) conducted a comprehensive meta-analysis confirming a strong and positive relationship between MT and AP, noting that this relationship is particularly pronounced in combat sports and individual sports. However, their analysis did not include MPS as an antecedent, nor did it test a mediation model. The meta-analysis synthesized existing correlational evidence but did not establish the pathways through which MT develops or influences performance.

To the best of our knowledge, no study has simultaneously tested MPS, MT, and AP within an integrated structural model in combat sports populations, and no study has specifically examined whether MT mediates the MPS-AP relationship using bootstrap-based mediation analysis. The present study addresses these gaps by: (a) testing the complete MPS → MT → AP pathway in combat athletes; (b) employing a combat-sport-specific athletic performance scale; and (c) using bootstrapping to rigorously test the significance of the indirect effect.

## 2 Theoretical framework and hypotheses

### 2.1 Conservation of resources (COR) theory as an overarching framework

The theoretical framework of this study is grounded in Conservation of Resources (COR) Theory (Hobfoll, 1989, 2002). COR theory posits that individuals strive to protect their existing psychological resources (e.g., attention, energy, self-confidence) and acquire new resources by investing in them. According to this theory, resource gain spirals occur when initial resource investment leads to resource accumulation, which in turn facilitates further resource acquisition (Hobfoll, 2002). Conversely, resource loss spirals can occur when individuals experience repeated or severe resource depletion without adequate replenishment, leading to increased vulnerability to stress and diminished psychological functioning.

In the context of combat sports, athletes face constant and unique stressors that differentiate their experience from many other sports. These include the risk of acute and chronic injury from physical contact, the pressure to maintain specific weight categories through often drastic weight-cutting practices, and intense competitive anxiety stemming from the direct, one-on-one nature of combat where performance outcomes are immediately visible and personally attributed (Mojtahedi et al., 2023; Lakicevic and Panfilova, 2025). These stressors collectively deplete psychological resources over time if not managed effectively.

MPS such as goal setting, imagery, and self-talk function as resource investment strategies that protect existing resources and generate new ones. According to Vealey (2024), mental skills are learnable psychological capabilities that help athletes optimize their performance and wellbeing. These skills include goal setting, imagery, self-talk, relaxation, and mindful practices, which athletes can develop systematically through mental training.

MT, in turn, represents a higher-order accumulated resource that emerges from the sustained and strategic use of these MPS. Rather than being an innate, fixed trait, MT is conceptualized within COR theory as a developable resource reservoir that grows through consistent investment. Once developed, MT serves as a buffer against stress-induced resource depletion, enabling athletes to withstand adversity, recover more quickly from setbacks, and maintain performance even when facing significant challenges (Hobfoll, 2002).

Recent empirical studies have confirmed that psychological resources enhance MT through COR-based mechanisms. Xu and Tian (2025) demonstrated a mediation model in which team cohesion influences MT through the sequential pathways of social support and self-identity, providing evidence for resource gain spirals in athletic contexts. Lakicevic and Panfilova (2025) critically examined the literature on combat sports and concluded that psychological skills training, when systematically applied, can enhance self-reported MT by targeting the cognitive and emotional resources that underpin resilience.

Thus, within the COR theory framework, the relationship among MPS, MT, and AP can be understood as a resource-based pathway: MPS serve as resource investment strategies that build and strengthen MT as an accumulated resource, which in turn facilitates the resource gain of AP. This study empirically tests this complete pathway in combat sports athletes.

### 2.2 Mental performance skills and mental toughness (H1)

MPS are defined as trainable psychological strategies including goal setting, imagery, self-talk, and relaxation, which athletes use to optimize their competitive state and perform under pressure (Vealey, 2024). From the perspective of COR theory, MPS function as resource investment strategies. According to the resource gain spiral principle, regular investment of psychological resources leads to resource accumulation (Hobfoll, 2002). Therefore, athletes who frequently use MPS are expected to develop higher levels of mental toughness, as MT represents an accumulated psychological resource.

Empirical evidence from multiple sources supports this relationship. A systematic review by Aditya et al. (2024) examined the effects of various psychological training programs on athletes and concluded that structured interventions such as mindfulness-based training and psychological skills training are effective in developing mental toughness. The review highlighted that athletes who regularly practice mental skills demonstrate greater psychological resilience when facing competitive pressure.

In the context of combat sports specifically, Sural et al. (2021) conducted a study with elite boxers and found that athletes who engaged in regular mental training practices reported substantially higher levels of MT compared to those who did not. The researchers emphasized that mental training should be considered a fundamental tool for developing MT, as the relationship between these two constructs was consistently strong across different levels of competition. More recently, Lakicevic and Panfilova (2025) provided a critical examination of MT in combat sports, noting that psychological skills training-particularly techniques such as imagery, relaxation strategies, and cognitive reappraisal-can enhance self-reported MT in combat athletes. They argued that unlike physical training alone, which primarily develops physiological capacity, structured psychological skills training directly targets the cognitive and emotional resources that underpin mental toughness.

Based on COR theory and the empirical evidence outlined above, the following hypothesis is proposed:

*H1*: MPS have a positive and significant effect on MT (MPS → MT).

### 2.3 Mental toughness and athletic performance (H2)

MT refers to the athlete’s capacity to maintain performance and recover despite pressure, stress, and adversity (Gucciardi et al., 2015). According to the resource gain spiral, accumulated resources facilitate further resource gain, ultimately leading to tangible outcomes such as performance (Hobfoll, 2002).

The MT-performance relationship has been extensively documented in the literature. A comprehensive meta-analysis by Hsieh et al. (2024) synthesized findings from multiple studies across different sports and found that the positive association between MT and AP is particularly strong in combat sports and individual sports. The researchers noted that this relationship becomes more pronounced as athletes mature, with adult athletes showing stronger MT-performance links compared to younger competitors.

In combat sports specifically, Slimani et al. (2016) examined kickboxing athletes and found that those with higher MT demonstrated superior performance outcomes across multiple physical and technical measures. The authors suggested that MT enables athletes to maintain focus and execute technical skills effectively even under the intense physical and psychological demands of competition.

Furthermore, Mojtahedi et al. (2023) investigated the role of MT in competitive anxiety among combat athletes. Their findings indicated that mentally tough athletes experience lower levels of competition-related anxiety, which in turn allows them to perform more consistently and effectively. The researchers concluded that MT serves as a protective psychological resource that helps athletes manage the inherent stressors of combat sports, thereby enhancing overall performance.

Based on COR theory and the empirical evidence outlined above, the following hypothesis is proposed:

*H2*: MT has a positive and significant effect on AP (MT → AP).

### 2.4 Mental performance skills and athletic performance (H3)

Beyond the indirect pathway through MT, MPS are expected to have a direct effect on AP. According to Vealey (2024), MPS directly optimize cognitive and emotional states during competition. Goal setting provides clear performance targets that guide behavior and effort; imagery enhances motor learning, confidence, and pre-competition preparation; self-talk helps regulate internal speech, reduce anxiety, and maintain focus; and arousal control allows athletes to achieve optimal physiological activation for peak performance (Birrer and Morgan, 2010).

Empirical evidence supports these direct effects. Bordo et al. (2025) conducted an eight-week multimodal mental intervention program with tennis players, which included relaxation exercises delivered via a smartphone application, variable training, and bi-weekly interviews. The results showed that athletes in the experimental group demonstrated notable improvements in self-confidence, awareness, arousal control, and refocusing skills, while their anxiety levels decreased significantly. These findings indicate that structured, technology-assisted mental preparation programs can be effective tools for improving athletes’ psychological skills and performance in a relatively short period.

In a study specifically focused on injury-related concerns, Duyan et al. (2025) found that athletes who engaged in regular mental training reported lower levels of injury anxiety and demonstrated enhanced optimal performance states. The researchers suggested that MPS help athletes maintain a positive focus and prevent the negative thought patterns that often accompany injury concerns, thereby supporting consistent performance.

A systematic review by Andreato et al. (2022), which focused specifically on combat sports, concluded that MPS consistently improve AP in combat athletes both before and after competitions. The review highlighted that mental skills training enhances technical and tactical execution, improves training efficiency, and supports psychological recovery following competitive efforts.

Based on COR theory and the empirical evidence outlined above, the following hypothesis is proposed:

*H3*: MPS have a positive and significant direct effect on AP (MPS → AP).

### 2.5 The mediating role of mental toughness (H4)

The most original and theoretically significant contribution of this study is the examination of MT’s mediating role in the relationship between MPS and AP. According to COR theory’s resource gain spiral, the pathway can be understood as follows: MPS function as resource investment strategies that protect and enhance existing psychological resources. Regular use of these strategies leads to resource accumulation, which manifests as the stable and resilient resource of MT. This accumulated resource then facilitates further resource gain in the form of athletic performance (Hobfoll, 2002). Thus, MT is expected to serve as a mediator that transmits the effects of MPS to AP.

The resource gain spiral mechanism can be illustrated as:

MPS (Resource Investment) → MT (Resource Accumulation) → AP (Resource Gain).

This theoretical prediction is supported by recent empirical findings. Xu and Tian (2025) conducted a study grounded in COR theory and demonstrated that psychological resources enhance MT through resource gain spirals. Their mediation model showed that psychological resources indirectly influence MT by first accumulating through supportive social and personal mechanisms. This finding provides direct empirical support for the notion that MT can function as a mediator between psychological resources and outcomes.

Lakicevic and Panfilova (2025) critically examined the literature on mental toughness in combat sports and concluded that while psychological skills training can enhance self-reported mental toughness, the specific mechanisms through which psychological skills translate into performance outcomes remain untested in combat sports populations. This gap in the literature underscores the need for research that explicitly examines how and why mental skills translate into performance gains via mental toughness.

The present study directly responds to this need by testing MT as a mediator between MPS and AP. Unlike previous studies that have examined these relationships in isolation or in pairwise combinations, this study tests the complete mediation pathway within a single integrated structural model using bootstrap-based mediation analysis.

Based on COR theory’s resource gain spiral and the empirical evidence outlined above, the following hypothesis is proposed:

*H4*: MT plays a mediating role in the relationship between MPS and AP (MPS → MT → AP).

## 3 Materials and methods

### 3.1 Research model

**Figure 1:**
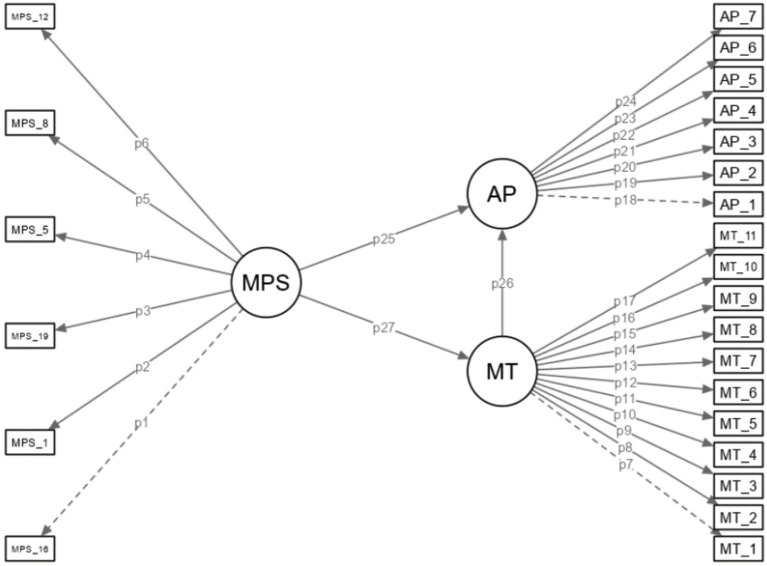
Research model. MPS, mental performance skill; MT, mental toughness; AP, athletic performance.

This study is a quantitative research based on a correlational survey model, aiming to examine the mediating role of MT in the relationship between MPS and AP in combat athletes. The correlational survey model is a research design intended to determine the level and direction of the relationship between two or more variables (Karasar, 2024). Structural Equation Modeling (SEM) was used to test the structural relationships among the variables. The hypothetical model of the research is shown in Figure 1.

### 3.2 Population and sample

The population of this study consists of licensed student-athletes actively competing in individual combat sports in university teams in Türkiye. The sample of the study comprises a total of 470 university combat sport athletes (243 male, 227 female) studying at various universities in the Türkiye, selected using convenience sampling. The following inclusion criteria were established for participant selection: (a) being 18 years of age or older, (b) having been regularly training in their combat sport for at least two years, (c) currently training regularly at least 3 days per week, and (d) being an active member of a university sports team.

The exclusion criteria were as follows: (a) having experienced a serious sports injury within the last six months, and (b) competing in a discipline other than the specified combat sports (Muay Thai, Kickboxing, Karate, Taekwondo, Judo).

### 3.3 Data collection process and sample adequacy

Prior to commencing the data collection phase of the study, ethical approval was obtained from the Social and Human Sciences Ethics Committee of Inönü University under protocol number 2026–33/4. The research was conducted in accordance with the ethical principles outlined in the Declaration of Helsinki.

To collect data, online survey links were sent to combat sport athletes in university teams via their coaches. The survey link was configured with IP address and cookie tracking to ensure that each participant completed the survey only once. A total of 517 survey links were distributed, and 496 responses were received (95.9% response rate).

After the data were organized, extreme values were identified based on skewness and kurtosis values. Following these criteria, data from a total of 26 participants (5.24%) were removed, and the final analyses were conducted using data from 470 participants. In the literature, it is stated that a data loss of 5–10% is acceptable in multivariate statistical analyses, particularly in assumption-sensitive techniques such as SEM (Tabachnick et al., 2013).

The required sample size for SEM was determined based on recommendations in the literature. Kline (2012) suggests that samples between 200 and 500 are generally sufficient depending on model complexity, while Tabachnick et al., 2013 state that samples above 300 yield reliable results. The total number of items across the scales used in this study was 28, and the sample size (*N* = 470) exceeded 10 times the number of items (Kline, 1994; Bryman and Cramer, 2001). Additionally, Comrey and Lee (2013) classify a sample size of 500 as “very good” in their sample size classification. Accordingly, the sample size was considered adequate for SEM analysis.

### 3.4 Self-report measures

#### 3.4.1 Mental performance skills scale (MPSS)

In this study, the MPS subscale of the “Sport Mental Training Questionnaire” developed by Behnke et al. (2017) and adapted into Turkish by Yarayan and İlhan (2018) was used. This subscale consists of items 1, 5, 8, 12, 16, and 19 and is scored using a 5-point Likert-type scale. The confirmatory factor analysis results reported in the Turkish adaptation study (Yarayan and İlhan, 2018) indicated that the scale had acceptable model fit (*χ*^2^/df = 1.85, GFI = 0.91, CFI = 0.95, NFI = 0.91, AGFI = 0.88). The factor loadings for the MPS subscale ranged between 0.62 and 0.78. Additionally, the Cronbach’s alpha internal consistency coefficient calculated for this subscale was found to be 0.85 (Yarayan and İlhan, 2018). These values are classified as ranging between “good” and “excellent” (Kline, 2005; George and Mallery, 2024). These findings support the validity and reliability of the measurement tool used.

#### 3.4.2 Mental toughness scale (MTS)

MT was assessed using an 11-item, single-dimensional scale developed by Madrigal et al. (2013) and adapted into Turkish by Erdoğan (2016). Participants rated the items on a 5-point Likert scale ranging from 1 (Strongly Disagree) to 5 (Strongly Agree). Higher total scores reflect greater mental toughness. The Cronbach’s alpha value for the Turkish adaptation of the scale was found to be 0.87 (Erdoğan, 2016).

#### 3.4.3 Athletic performance scale (APS)

The Athletic Performance Scale developed by Bingöl and Yildiz (2021) was used to determine the performance of combat athletes. The scale consists of a single dimension and 7 items. The single-factor structure of the scale was tested in a sample of elite athletes who had achieved various degrees in combat sports (Boxing, Muay Thai, Kickboxing, Wushu, and Taekwondo) in Türkiye, and it was concluded that the single-factor structure was confirmed. All scale items were measured using Likert-type intervals (1 = Never; 7 = Always). As a result of the validity and reliability studies, the suitability of the scale for factor analysis was examined using the Kaiser-Meyer-Olkin (KMO) and Bartlett’s Sphericity tests. The KMO value obtained from the test was 0.824, which is considered excellent, and the Bartlett’s Sphericity test result was significant (*χ*^2^ = 678.369; df = 21; *p* = 0.000). The factor loadings in the single dimension ranged between 0.548 and 0.786. All these values indicate that the scale is suitable for factor analysis. The Cronbach’s alpha internal consistency reliability coefficient was found to be 0.81. Therefore, these values indicate that the scale is “highly reliable” (Bingöl and Yildiz, 2021).

### 3.5 Data analysis

The data of the study were analyzed using the Jamovi 2.7.6 statistical package program. The skewness and kurtosis values for all variables were within the range of −1.96 to 1.96 (George and Mallery, 2010), indicating that the data met the assumptions of univariate normality. Although the data satisfied the normality assumption, parameter estimation in the structural equation model was performed using the robust weighted least squares (WLSMV) method, as the scales used in the study were Likert-type (ordinal) in nature. This method provides more reliable results, particularly for ordinal data structures (Flora and Curran, 2004; Brown, 2015; Li, 2016).

The relationships among the variables were tested using Pearson correlation analysis. The construct validity of the scales was examined through Confirmatory Factor Analysis (CFA); model fit was assessed using the χ^2^/df ratio, RMSEA, CFI, TLI, NFI, and SRMR indices. For reliability, Cronbach’s *α* was used; for convergent validity, AVE and CR were used; for discriminant validity, the Fornell-Larcker criterion and the HTMT ratio were used.

SEM was preferred over alternative methods (e.g., multiple regression or PROCESS macro) because it accounts for measurement error by using latent variables, allows simultaneous testing of multiple direct and indirect paths, and provides model fit statistics to evaluate the overall theoretical structure (Kline, 2023).

Structural Equation Modeling (SEM) was employed to test the research hypotheses. The significance of indirect effects was tested by calculating 95% confidence intervals using 5.000 bootstrap samples. As a result of bootstrapping, the indirect effect was considered significant if the lower (BootLLCI) and upper (BootULCI) confidence interval values did not include zero (Hayes, 2018). The significance level was set at *p* < 0.05.

Following contemporary mediation analysis practices (Hayes, 2018), we focus on the significance of the indirect effect (a × b) rather than requiring individual paths to be statistically significant. In this study, path a represents the effect of MPS on MT, path b represents the effect of MT on AP, and the indirect effect (a × b) represents the effect of MPS on AP through MT. The indirect effect is considered significant if the bias-corrected bootstrap confidence interval does not contain zero, regardless of the significance of the direct effect or the individual paths. The total effect (c) is decomposed into the direct effect (c′) and the indirect effect (ab): c = c′ + ab.

## 4 Results

### 4.1 Descriptive statistics

The population of this study consists of athletes engaged in individual combat sports within university teams in Türkiye. The sample comprises 470 university-level combat sports athletes, selected from various universities across Türkiye using a convenience sampling method and who participated voluntarily. Of the participants, 51.7% (*n* = 243) were male and 48.3% (*n* = 227) were female. Regarding age distribution, 51.3% (*n* = 241) were in the 22–25 age range, 30.2% (*n* = 142) were 26 years and older, and 18.5% (*n* = 87) were in the 18–21 age range. In terms of sports discipline distribution, Muay Thai had the highest percentage at 42.1% (*n* = 198), followed by Kickboxing (26.8%, *n* = 126), Taekwondo (14.5%, *n* = 68), Karate (11.7%, *n* = 55), and Judo (4.9%, *n* = 23). Regarding total sports experience, 88.3% (*n* = 415) of participants had 7 years or more, 8.1% (*n* = 38) had 6–7 years, 2.3% (*n* = 11) had 4–5 years, and 1.3% (*n* = 6) had 2–3 years of experience. Additionally, 67.7% (*n* = 318) of the participants held national athlete status. Detailed information regarding the demographic and sports-related characteristics of the sample is summarized in Table 1.

**Table 1:** Demographic characteristics of the athletes.

Variables		f	%
Gender	Male	243	51.7
	Female	227	48.3
	Total	470	100.0
Age	18–21 years	87	18.5
	22–25 years	241	51.3
	26 years and above	142	30.2
	Total	470	100.0
Sports discipline	Judo	23	4.9
	Karate	55	11.7
	Taekwondo	68	14.5
	Muaythai	198	42.1
	Kickbox	126	26.8
	Total	470	100.0
National athlete	Yes	318	67.7
	No	152	32.3
	Total	470	100.0
Total sports experience	2–3 years	6	1.3
	4–5 years	11	2.3
	6–7 years	38	8.1
	7 years and above	415	88.3
	Total	470	100.0

### 4.2 Common method Bias

To assess the potential threat of common method bias, Harman’s single-factor test was conducted. All items from the Mental Performance Skills Scale, the Mental Toughness Scale, and the Athletic Performance Scale were entered into an unrotated exploratory factor analysis. The results revealed that a single factor accounted for 32.6% of the total variance, which is well below the recommended threshold of 50% (Podsakoff et al., 2003). This indicates that common method bias does not pose a significant threat to the validity of the study’s findings.

### 4.3 Descriptive statistics, pearson correlation and discriminant validity (Fornell-Larcker, HTMT)

The means, standard deviations, skewness, and kurtosis values for the variables, along with the Pearson correlation analysis results and discriminant validity findings, are presented in Table 2.

**Table 2:** Descriptive statistics, pearson correlation, and discriminant validity (fornell-larcker, HTMT).

Variables	M	SD	Skewness	Kurtosis	1	2	3
1. MPS	23.1	4.41	−0.413	−0.170	**(0.707)**	0.394***	0.422***
2. MT	48.5	6.16	−1.120	1.940	**0.496**	**(0.787)**	0.445***
3. AP	29.5	4.72	−1.160	1.890	**0.598**	**0.543**	**(0.781)**

M, Mean; SD, Standard deviation; MPS, Mental performance skills; MT, Mental toughness; AP, Athletic performance. Values in parentheses on the diagonal are the square roots of the AVE (Fornell-Larcker criterion). Values below the diagonal in bold are the HTMT ratios. Values above the diagonal are the Pearson correlation coefficients. ***p < 0.001. All HTMT values are below 0.85, confirming discriminant validity.

When the descriptive statistics were examined, the mean for MPS was 23.1 (SD = 4.41), the mean for MT was 48.5 (SD = 6.16), and the mean for AP was 29.5 (SD = 4.72). An examination of the skewness and kurtosis values revealed that the skewness values for all variables ranged between −1.160 and −0.413, while the kurtosis values ranged between −0.170 and 1.940. These values falling within the ±2 range recommended by George and Mallery (2010) indicates that the data satisfied the assumption of normal distribution.

According to Cohen (2013) conventions for effect sizes (*r* = 0.10 small, *r* = 0.30 medium, *r* = 0.50 large), the correlation between MPS and MT (*r* = 0.394) represents a medium-to-large effect, while the correlations between MPS and AP (*r* = 0.422) and between MT and AP (*r* = 0.445) are also medium-to-large. These effect sizes indicate that the shared variance among constructs ranges from approximately 15.5 to 19.8%, suggesting practically meaningful relationships.

Within the scope of discriminant validity, the Fornell-Larcker criterion and HTMT ratios were examined. According to the Fornell-Larcker criterion, the square root of the AVE for each construct (MPS = 0.707; MT = 0.787; AP = 0.781) was greater than the correlations of the respective construct with other constructs, thus discriminant validity was established (Fornell and Larcker, 1981). Additionally, the HTMT values (MPS-MT = 0.496; MPS-AP = 0.598; MT-AP = 0.543) being below the recommended threshold of 0.85 further supports discriminant validity (Henseler et al., 2015).

### 4.4 Confirmatory factor analysis results

The confirmatory factor analysis (CFA) results revealed that the measurement model demonstrated acceptable fit. The analysis showed that the item factor loadings for MPS ranged between *β* = 0.525 and *β* = 0.843, for mental toughness MT ranged between *β* = 0.712 and *β* = 0.817, and for AP ranged between *β* = 0.719 and *β* = 0.876. All factor loadings were statistically significant (*p* < 0.001). The factor loadings, reliability, and validity values for the measurement model are presented in Table 3.

**Table 3:** Factor loadings, reliability and validity of the measurement model.

Latent	Observed	*β*	SE	z	*p*	LLCI (95%)	ULCI (95%)	CR	AVE	Cronbach’s *α*
MPS	MPS_1	0.796	0.0000			1.000	1.000	0.820	0.500	0.769
	MPS_5	0.843	0.0404	26.2	<0.001	0.980	1.138			
	MPS_8	0.804	0.0389	26.0	<0.001	0.933	1.086			
	MPS_12	0.538	0.0453	14.9	<0.001	0.587	0.764			
	MPS_16	0.664	0.0512	16.3	<0.001	0.733	0.934			
	MPS_19	0.525	0.0498	13.2	<0.001	0.561	0.757			
MT	MT_1	0.712	0.0000			1.000	1.000	0.916	0.620	0.896
	MT_2	0.759	0.0463	23.0	<0.001	0.975	1.156			
	MT_3	0.805	0.0461	24.5	<0.001	1.040	1.221			
	MT_4	0.809	0.0487	23.3	<0.001	1.041	1.232			
	MT_5	0.806	0.0452	25.0	<0.001	1.043	1.220			
	MT_6	0.813	0.0429	26.6	<0.001	1.057	1.225			
	MT_7	0.797	0.0481	23.3	<0.001	1.025	1.213			
	MT_8	0.817	0.0426	26.9	<0.001	1.063	1.230			
	MT_9	0.795	0.0487	22.9	<0.001	1.021	1.211			
	MT_10	0.800	0.0496	22.6	<0.001	1.025	1.219			
	MT_11	0.738	0.0488	21.2	<0.001	0.940	1.131			
AP	AP_1	0.719	0.0000			1.000	1.000	0.879	0.610	0.867
	AP_2	0.766	0.0474	22.5	<0.001	0.973	1.159			
	AP_3	0.876	0.0484	25.2	<0.001	1.124	1.314			
	AP_4	0.834	0.0492	23.6	<0.001	1.064	1.256			
	AP_5	0.768	0.0448	23.9	<0.001	0.981	1.157			
	AP_6	0.719	0.0471	21.2	<0.001	0.907	1.092			
	AP_7	0.771	0.0500	21.4	<0.001	0.974	1.170			

β, Standardized factor loading; SE, Standard error; LLCI, Lower level confidence interval; ULCI, Upper level confidence interval; CR, Composite reliability; AVE, Average variance extracted; α, Cronbach’s alpha reliability coefficient. All factor loadings are significant at the *p* < 0.001.

The model fit indices were calculated as *χ*^2^/sd = 4.08, CFI = 0.983, TLI = 0.981, NFI = 0.977, RMSEA = 0.08, and SRMR = 0.07. According to Kline (2023), CFI, TLI, and NFI values above 0.95 indicate excellent fit, while RMSEA values between 0.05 and 0.08, SRMR values ≤ 0.08, and *χ*^2^/sd values between 2 and 5 indicate acceptable fit. Accordingly, in our study, *χ*^2^/sd (4.08), RMSEA (0.08), and SRMR (0.07) fall within the range of acceptable fit, while CFI (0.983), TLI (0.981), and NFI (0.977) indicate excellent fit.

As presented in Table 3, all constructs met the recommended thresholds for reliability (Cronbach’s *α* ≥ 0.70), convergent validity (AVE ≥ 0.50), and composite reliability (CR ≥ 0.70) (Fornell and Larcker, 1981; Hair et al., 2019).

### 4.5 Mediation analysis results

The path analysis results regarding the relationships among MPS, MT, and AP in combat athletes are presented in Table 4.

**Table 4:** Path analysis results with bootstrap confidence intervals.

Dep	Pred	Estimate	SE	LLCI (95%)	ULCI (95%)	*β*	z	*p*
Direct effects								
AP	MPS	0.319	0.0384	0.244	0.394	0.353	8.30	<0.001
AP	MT	0.365	0.0448	0.277	0.452	0.361	8.14	<0.001
MT	MPS	0.432	0.0401	0.353	0.510	0.483	10.78	<0.001
Indirect effect								
MPS ⇒ MT ⇒ AP		0.157	0.024	0.111	0.204	0.174	6.649	<0.001

Dep, Dependent variable; Pred, Predictor variable; MPS, Mental performance skills; MT, Mental toughness; AP, Athletic performance; B, unstandardized regression coefficient (Estimate); SE, standard error; LLCI, Lower level confidence interval; ULCI, Upper level confidence interval; β, standardized regression coefficient. The indirect effect and all confidence intervals are based on 5,000 bootstrap samples.

The indirect effect was tested using the bootstrap method (5,000 resamples). Following Hayes (2018), we focus on the significance of the indirect effect (a × b) rather than requiring individual paths to be statistically significant. In this study, path a represents the effect of MPS on MT (*β* = 0.483, *p* < 0.001), path b represents the effect of MT on AP (*β* = 0.361, *p* < 0.001), and the indirect effect (a × b) represents the effect of MPS on AP through MT.

Bootstrap analysis revealed that MPS had a positive and statistically significant indirect effect on AP through MT (indirect effect: a × b = 0.157, SE = 0.024, 95% CI [0.111, 0.204]; standardized indirect effect *β* = 0.174, *p* < 0.001). The indirect effect is considered significant because the bias-corrected bootstrap confidence interval does not contain zero (Hayes, 2018), regardless of the significance of the direct effect or the individual paths.

**Figure 2:**
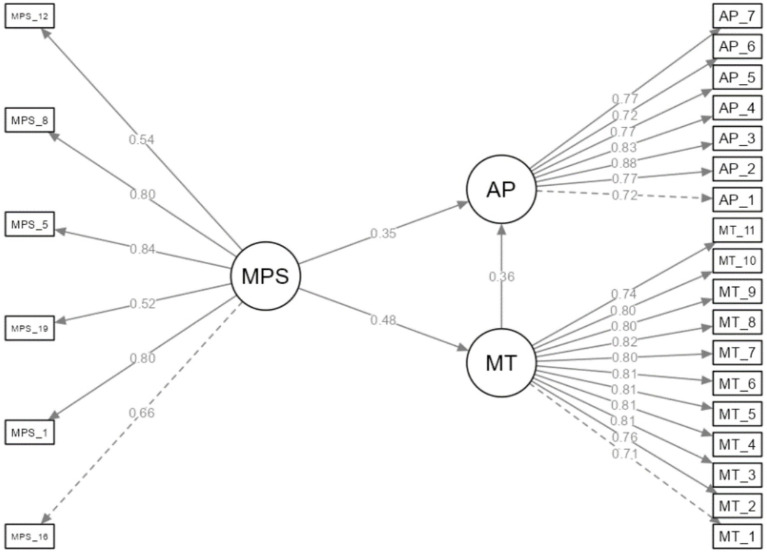
SEM analysis results. MPS, Mental performance skills; MT, mental toughness; AP, athletic performance.

The total effect (c) is decomposed into the direct effect (c′) and the indirect effect (ab): c = c′ + ab. In this study, the direct effect of MPS on AP (c′) was *β* = 0.353 (*p* < 0.001), and the indirect effect (ab) was *β* = 0.174 [95% CI (0.111, 0.204)]. Thus, MPS influence AP both directly and indirectly through MT. The tested SEM is presented in Figure 2.

## 5 Discussion

This study aimed to examine the relationship of MPS on AP in combat athletes and the mediating role of MT in this relationship. According to the pearson correlation analysis results, positive and statistically significant relationships were found among all variables (*p* < 0.001). Significant positive relationships were found between MPS and MT (*r* = 0.394, *p* < 0.001), between MPS and AP (*r* = 0.422, *p* < 0.001), and between MT and AP (*r* = 0.445, *p* < 0.001).

When examining the findings regarding the direct effects tested within the SEM, MPS were found to be positive and significant predictors of both AP (*β* = 0.353, *p* < 0.001) and MT (*β* = 0.483, *p* < 0.001). Additionally, MT was found to be a significant predictor of AP (*β* = 0.361, *p* < 0.001). The findings generally support the hypotheses structured within the framework of Conservation of Resources Theory (Hobfoll, 1989) and contemporary sport psychology. The analyses revealed that MPS have both a direct and an indirect effect on AP through MT. We aim to provide a comprehensive evaluation of the psychological skills and strategies of combat athletes in order to assist coaches and sport psychologists in the better preparation and implementation of mental training.

### 5.1 The effect of MPS on AP (H1)

The first of the study’s main hypotheses was that MPS would have a significant and positive direct effect on AP in combat athletes. Both the correlation (*r* = 0.422) and the structural model analysis (*β* = 0.353, *p* < 0.001) supported this hypothesis. According to Conservation of Resources Theory (Hobfoll, 1989), mental skills such as goal setting, imagery, and focus are strategic tools that protect the athlete’s limited psychological energy resources and effectively direct these resources toward performance. MPS are a fundamental psychological tool that enables athletes to effectively cope with sporting pressures. The foundation of mental training is based on Cognitive-Behavioral Theory, which focuses on regulating internal speech and cognitive processes aimed at increasing attention and concentration, and reducing stress and anxiety (Harshitha and Komala, 2025). For instance, Bordo et al. (2025) conducted an 8-week multimodal mental intervention program (including variable training, relaxation exercises delivered via a smartphone application, and bi-weekly interviews) with tennis players. The results showed a statistically significant increase in self-confidence, arousal control, awareness, and refocusing skills, and a significant decrease in anxiety levels in the experimental group. In contrast, no significant changes were observed in these variables over time in the control group. These findings indicate that a structured, technology-assisted mental preparation program can be an effective tool for improving athletes’ psychological skills and performance in a short period.

Various studies have shown that mindfulness-based interventions improve psychological skills in athletes (Mehrsafar et al., 2019; Jones et al., 2020; Terzioğlu et al., 2020). These studies reveal that mindfulness training leads to significant improvements in concentration, self-confidence, goal setting, stress management, and coping skills in athletes. Findings have also shown that structured mindfulness training is an effective tool for strengthening athletes’ psychological performance resources (Kelemen et al., 2025). Research indicates that MPS training, such as goal setting, imagery, self-talk, relaxation, and mindfulness, improves psychological preparation and enhances sport performance (Urfa and Aşçı, 2018; Filik Gül and Uz Baş, 2024; Erdoğan Yüce et al., 2025; Harshitha and Komala, 2025).

Recent studies have found that athletes with higher sport performance possess stronger imagery skills (Demir et al., 2025; Volgemute et al., 2025). For example, Duyan et al. (2025) found in a study conducted with athletes that MPS reduced injury anxiety and enhanced optimal performance. According to a systematic review conducted specifically on combat athletes, MPS have been reported to improve AP in combat sports athletes before and after competitions (Andreato et al., 2022). Mental skills training has been shown to significantly enhance athletic performance, reduce anxiety, support psychological resilience, and promote peak performance and long-term well-being (Harshitha and Komala, 2025). Therefore, it can be stated that MPS are an important psychological tool that can enhance the technical and tactical skills and training efficiency of combat athletes.

### 5.2 The effect of MPS on MT (H2)

The second important finding of the study is that MPS have a positive and significant predictive power on MT (*β* = 0.483, *p* < 0.001). Among the interventions aimed at developing MT, mindfulness and psychological skills training stand out. For example, a systematic review conducted by Aditya et al. (2024) found that such training programs significantly increased athletes’ MT levels. This finding supports the effectiveness of mindfulness-based approaches and psychological skills training in sport psychology practices. Demir et al. (2025) found in a study conducted with elite athletes that athletes with high MT also had high levels of imagery. Soundara Pandian et al. (2023), in their narrative synthesis review, demonstrated that MT is a developable capacity and highlighted its important role in performance. Notably, it was emphasized that structured interventions such as psychological skills training, mindfulness, imagery, and relaxation techniques are effective in strengthening MT, whereas physical training alone does not produce the same effect (Soundara Pandian et al., 2023). Sural et al. (2021), in their study conducted with elite boxers, found that athletes’ mental training levels significantly predicted their MT levels. The model explained approximately 34% of the variance in MT (*R*^2^ = 0.34). This finding indicates that an increase in mental training practices is associated with a statistically significant improvement in MT, and therefore mental training should be considered a fundamental tool for developing MT.

This finding can be meaningfully interpreted within the framework of Conservation of Resources Theory (Hobfoll, 1989). According to the theory, individuals strive to protect their valuable resources (attention, energy, self-confidence) and acquire new resources. In this context, MPS (goal setting, imagery, self-talk) function as strategic tools that protect the athlete’s existing psychological resources and invest in them. The regular and effective use of these strategies leads to the accumulation of a more stable and resilient resource, such as mental toughness, consistent with the “resource gain spiral” proposed by Conservation of Resources Theory. Therefore, the association between increased mental training practices and a statistically significant improvement in MT levels is consistent with theoretical expectations.

### 5.3 The effect of MT on AP (H3)

The third significant finding of the study is that MT is a significant and positive predictor of AP (*β* = 0.361, *p* < 0.001). MT has positive effects on AP by enhancing athletes’ ability to cope with stress, pressure, and challenging conditions (Răzvan et al., 2021; Wheatley et al., 2023; Aditya et al., 2024). However, the magnitude of the MT-performance relationship can vary significantly depending on variables such as sport type, athlete’s age group, and the performance measurement method used. A review covering the years 2000–2020 clearly revealed these contextual differences. Although a positive association between MT and performance was found in most of the studies evaluated in that review, this association was not statistically significant in certain sports such as equestrian and alpine skiing, while in basketball, it was identified as a strong predictor rather than a mere correlation (Guszkowska and Wojcik, 2021). The meta-analytic findings of Hsieh et al. (2024) revealed that the MT–performance relationship is much stronger, particularly in combat sports (*r* = 0.73) and individual sports (*r* = 0.73). Additionally, this relationship was found to be more pronounced in adult athletes (*r* = 0.41) compared to adolescent athletes (*r* = 0.20). Combat sports such as judo, karate, and taekwondo provide individuals interested in combat sports with an effective context for stress management, emotional regulation, and control of aggressive impulses (Oh et al., 2025). Tao and Li (2025) found that university students with martial arts experience exhibited higher levels of MT and self-control compared to students without such experience. An experimental study found that a 10-week martial arts-based intervention increased the psychological resilience of middle school students aged 12–14 (Moore et al., 2021). A study conducted on kickboxing athletes found a strong overall correlation between MT and muscle strength performance (Slimani et al., 2016). Therefore, it can be said that it is critically important for sports professionals to effectively develop MT to enhance AP and well-being (Wheatley et al., 2023).

### 5.4 The mediating role of MT and the holistic evaluation of the model (H4)

The most original and theoretically significant contribution of this study is that the mediating role of MT in the relationship between MPS and AP was confirmed through bootstrap analysis (indirect effect: a × b = 0.157, SE = 0.024, 95% CI [0.111, 0.204]; standardized indirect effect *β* = 0.174, *p* < 0.001). Based on this finding, it is demonstrated that MPS are associated with AP by strengthening combat athletes’ mental resilience against stress and pressure. MT is defined as possessing a natural or developed psychological advantage that enables athletes to cope better than their opponents with the demands and pressures of the highest levels of sports competition (Bíró and Balogh, 2025). Stoyanova et al. (2025) explain this concept through two fundamental theoretical models: the Hardiness Model (Kobasa et al., 1982a, 1982b) and Cognitive Appraisal Theory (Lazarus and Folkman, 1984). According to the Hardiness Model, MT mitigates the negative effects of stress and supports adaptive responses through the dimensions of commitment (active engagement with life), control (belief in one’s ability to influence events), and challenge (viewing change or difficulty as an opportunity rather than a threat) (Kobasa et al., 1982a, 1982b). Cognitive Appraisal Theory, on the other hand, suggests that hardiness stems from the processes of primary appraisal (interpreting stressors as a challenge rather than a threat) and secondary appraisal (believing that one has adequate coping resources) (Lazarus and Folkman, 1984). Athletes regularly face numerous challenges that elevate stress levels and can potentially hinder optimal performance. It can be said that MT is considered a crucial factor for athletic success because it helps athletes manage physical and psychological demands (Dziuba et al., 2025). Khodabandelou and Salehian (2023) state that enhancing athletes’ psychological resilience and self-efficacy levels can positively affect their performance. Increased self-efficacy enables athletes to cope more effectively with negative psychological factors that impede performance and to contend with the challenges they face. Bojkowski (2026) proposes that MT fosters a positive mental attitude by nurturing constructive cognitive strategies, and that this process serves a protective function against athlete burnout. This protective mechanism integrates psychological resources such as self-confidence and competence into a coherent system, enabling the athlete to maintain commitment and motivation even in the face of failure and adversity. Zhong et al. (2025) present a framework for helping athletes cope with the intense pressures of training and competition. The researchers argue that mindfulness meditation can strengthen athletes’ cognitive reappraisal skills and MT, helping them respond more resiliently to stressful situations and optimize their performance even under adverse conditions. Furthermore, when negative psychological experiences occur, it is critically important to recognize them early and adopt proactive coping methods such as strategic rest, seeking social support, communicating with coaches, or seeking professional help. It is noted that normalizing psychological difficulties and intervening in a timely manner is important for safeguarding an athlete’s long-term performance and well-being. Therefore, based on all these findings, it is evident that our H_4_ hypothesis is consistent with the literature.

Although our findings support the mediating role of MT, alternative explanations should be considered. One possibility is reverse causality: athletes with higher baseline MT may be more likely to engage in MPS practice, rather than MPS causing increased MT. This bidirectional possibility has been noted in sport psychology research (Nicholls et al., 2016). Longitudinal studies are needed to establish the temporal precedence of MPS over MT.

Another alternative is that unmeasured third variables, such as coach-athlete relationship quality or motivational climate, could influence both MPS and MT. For instance, Habeeb et al. (2023) found that coach-initiated mastery climate indirectly influenced athlete outcomes through peer-initiated climate, suggesting that social-environmental factors may play a role in shaping psychological resources. Future research should examine whether variables such as coaching style or team climate moderate or confound the MPS-MT-AP relationship.

### 5.5 Theoretical and practical implications

#### 5.5.1 Theoretical implications

The findings of this study test fundamental theoretical models in the field of sport psychology within the context of combat sports, thereby making important contributions to understanding how these models operate in environments characterized by high stress and instantaneous decision-making. The results obtained are consistent with the Hardiness Model (Kobasa et al., 1982a, 1982b), Cognitive Appraisal Theory (Lazarus and Folkman, 1984), and Conservation of Resources Theory (Hobfoll, 1989). Within the framework of Conservation of Resources Theory, MPS (goal setting, imagery, self-talk) function as strategies that protect and invest in the athlete’s limited psychological resources (attention, energy, self-confidence). The effective use of these strategies leads to the accumulation of a more stable and resilient resource known as MT. This process exemplifies the “resource gain spiral” posited by Conservation of Resources Theory: the protection and utilization of existing resources MPS enables the acquisition of new and stronger resources MT.

The mediating role of MT empirically demonstrates how, as these theories predict, the processes of reframing challenges as “challenge” rather than “threat” (primary appraisal) and the individual’s belief in their control and coping resources (secondary appraisal) mediate performance. This finding reinforces the validity of these theories in the context of competitive sports, particularly in combat sports where performance is shaped at the intersection of physical and cognitive skills.

By moving beyond traditional approaches that directly link MPS (goal setting, imagery, self-talk) to performance outcomes, this study reveals the mediating role of MT in this relationship. This finding underscores the importance of understanding how these skills nourish the athlete’s overall psychological resilience system, rather than focusing solely on technical skills or isolated psychological techniques. The integrated pathway model of “MPS → MT → AP” enriches existing models in the literature specifically for combat sports and sheds light on the transformation process of psychological resources.

As emphasized by Bojkowski (2026) and Khodabandelou and Salehian (2023), the research results demonstrate that MT is not a standalone trait; rather, it shows how various psychological resources such as self-efficacy, positive mental attitude, and constructive cognitive strategies are integrated within a protective and developmental system. This supports theoretical approaches that conceptualize athlete resilience as a “resource pool” and empirically confirms the protective function of MT against burnout.

While our findings support COR theory as an overarching framework, other theoretical perspectives may also explain the MPS-MT-AP relationship. SDT (Deci and Ryan, 2000) suggests that satisfaction of basic psychological needs (autonomy, competence, relatedness) fosters intrinsic motivation and psychological well-being. From an SDT perspective, MPS may enhance MT by satisfying athletes’ need for competence through mastery experiences and goal attainment. Similarly, the Broaden-and-Build Theory of Positive Emotions (Fredrickson, 2001) proposes that positive emotions broaden cognitive and behavioral repertoires, building enduring personal resources such as resilience and MT. Future research could integrate these complementary frameworks to provide a more comprehensive understanding of the psychological mechanisms underlying athletic performance in combat sports.

#### 5.5.2 Practical implications

The research results offer evidence-based practice strategies for coaches, sport psychologists, and athletes:

First, the finding that MPS (goal setting, imagery, self-talk, mindfulness) nurture mental toughness indicates that these skills should be systematically integrated into physical training routines. Training programs should be structured not only to develop physical capacity but also to cultivate the psychological mindset (belief in control and challenge) needed to utilize this capacity under stress. Particularly for combat athletes, alongside technical training, regular imagery, breathing exercises, and focus work should become an integral part of training periodization.

Second, in alignment with Zhong et al. (2025), interventions such as mindfulness meditation are seen as effective tools for strengthening athletes’ cognitive reappraisal skills and, consequently, their mental toughness. Such practices should be incorporated, especially into pre-competition anxiety management and post-competition recovery processes. Clubs can enhance athletes’ stress management skills by adding brief mindfulness sessions to their weekly training programs.

Third, normalizing psychological difficulties and emphasizing early intervention is critically important. Sports clubs should establish regular psychological screening and counseling services centered on confidentiality and trust for athletes; athletes should be encouraged to share negative emotions, communicate openly with coaches, and seek professional support when needed.

Fourth, considering the protective effect of MT against burnout (Bojkowski, 2026), it is clear that this construct is vital not only for performance but also for the athlete’s long-term health and career sustainability. Therefore, mental toughness training should be incorporated into the development programs of young athletes from an early age, and for athletes approaching retirement, guidance should be provided on transferring this mental strength to post-sport life.

Fifth, for athletes with relatively low levels of MT, targeted programs focusing specifically on components such as emotional control, self-confidence, and stress management should be designed as part of psychological skills training. Personalized psychological preparation protocols should be developed considering age group, sport discipline, and individual needs.

Sixth, the positive results of the 8-week multimodal mental intervention program conducted by Bordo et al. (2025) with tennis players demonstrate the effectiveness of technology-assisted (smartphone application) mental training practices. Developing similar digital platforms for combat athletes could enable them to develop their psychological skills outside of training and competition.

### 5.6 Limitations and suggestions for future research

The findings of this study should be evaluated within the context of certain methodological limitations. First, the cross-sectional research design does not allow for establishing causality between variables, permitting only correlational inferences. Second, the fact that all data were collected via self-report scales carries the risk of common method variance and social desirability bias. A third limitation is that performance measurement was confined to a subjective scale; the absence of additional measures such as objective competition statistics or coach evaluations may limit the scope of the performance variable. Fourth, there are a number of factors that could influence the relationships in the model and were not controlled for (e.g., athlete’s years of experience, coach-athlete relationship quality, team climate, personality traits, Social support). Finally, the sample consisting solely of university-level combat athletes in Türkiye limits the generalizability of the findings to other cultures, age groups, or elite professional athletes.

These limitations also provide valuable directions for future research. The priority for future studies should be to employ randomized controlled experimental designs or longitudinal designs to test the causal effect of MPS training on MT and AP. A second recommendation is to adopt a multi-method approach to reduce self-report bias and enhance the validity of findings, integrating coach evaluations, objective performance records, and physiological indicators measuring stress response into the research design. Furthermore, the current model could be enriched with variables that better explain the source of the relationships (e.g., satisfaction of basic psychological needs, coach leadership styles), and the moderating roles of factors such as gender, sport discipline, or competition level could be tested to examine for which groups the model is stronger. Finally, the cross-cultural and cross-disciplinary validity of the proposed path model should be tested by examining it in different cultural contexts and in other disciplines such as team or endurance sports, in addition to combat sports, to assess the generalizability of the theoretical framework.

Finally, future research should examine potential moderators that may influence the strength of the observed relationships. For instance, sport type (individual vs. team) could moderate the MPS-MT-AP pathway, as previous meta-analytic findings suggest that the MT-performance relationship is stronger in individual sports compared to team sports (Hsieh et al., 2024). Similarly, competitive level (amateur vs. elite) and age group (adolescent vs. adult) may influence the magnitude of the mediation effect (Hsieh et al., 2024). Multi-group SEM or moderated mediation analysis could be employed to test these potential moderators.

## 6 Conclusion

This study demonstrated that MPS are associated with AP both directly and indirectly by strengthening mental toughness. These findings make several contributions to the sport psychology literature.

First, the study tests the proposed mediating mechanism in combat athletes, responding to recent calls in the literature for examining how psychological factors influence performance in high-stress sports. Second, by confirming the mediating role of mental toughness, the study shows that mental toughness is not merely a performance correlate but an active psychological mechanism that translates trainable mental skills into performance outcomes. Third, the findings extend Conservation of Resources Theory to combat sports, providing empirical support for the resource gain spiral in this specific athletic context.

In conclusion, structured mental skills training and programs aimed at developing mental toughness can improve not only competition results but also fundamental psychological capacities such as stress management and coping with challenging conditions. Creating a training environment where coaches and sport psychologists systematically develop athletes’ cognitive and emotional skills and integrate strategies that foster mental toughness can enhance both psychological resilience and performance in combat athletes. Optimal performance is achievable through a holistic approach where physical preparation and mental toughness reinforce each other.

## Data Availability

The raw data supporting the conclusions of this article will be made available by the authors, without undue reservation.
